# Availability, prices and affordability of the World Health Organization’s essential medicines for children in Guatemala

**DOI:** 10.1186/1744-8603-8-22

**Published:** 2012-07-02

**Authors:** Angela Anson, Brooke Ramay, Antonio Ruiz de Esparza, Lisa Bero

**Affiliations:** 1 Department of Clinical Pharmacy, University of California, San Francisco, CA 94143-0622, USA; 2 La Universidad del Valle de Guatemala, Vista Hermosa III, Guatemala City, Guatemala 01015; 3 Cedar Associates, Menlo Park, CA 94025, USA; 4 Department of Clinical Pharmacy, Institute for Health Policy Studies, University of California, San Francisco, CA 94118, USA

**Keywords:** Guatemala, Essential medicines for children, National formulary, Availability, World Health Organization

## Abstract

**Background:**

Several World Health Organization (WHO) initiatives aim to improve the accessibility of safe and effective medicines for children. A first step in achieving this goal is to obtain a baseline measure of access to essential medicines. The objective of this project was to measure the availability, prices, and affordability of children’s medicines in Guatemala.

**Methods:**

An adaption of the standardized methodology developed by the World Health Organization and Health Action International (HAI) was used to conduct a cross sectional survey to collect data on availability and final patient prices of medicines in public and private sector medicine outlets during April and May of 2010.

**Results:**

A subset of the public sector, Programa de Accesibilidad a los Medicamentos (PROAM), had the lowest average availability (25%) compared to the private sector (35%). In the private sector, highest and lowest priced medicines were 22.7 and 10.7 times more expensive than their international reference price comparison. Treatments were generally unaffordable, costing as much as 15 days wages for a course of ceftriaxone.

**Conclusions:**

Analysis of the procurement, supply and distribution of specific medicines is needed to determine reasons for lack of availability. Improvements to accessibility could be made by developing an essential medicines list for children and including these medicines in national purchasing lists.

## Background

Children in poorer countries are more likely to die from treatable conditions than those in higher resource settings because they do not receive appropriate or timely medications [1]. This is also true in Guatemala, where poverty contributes to the country’s high mortality rate for children under the age of five [2]. Millennium Development Goal 4 (MDG4) states that a two-thirds reduction in mortality from 1990 to 2015 in children younger than five years is essential for global development [3]. Compared to the rate of mortality in 1990, Guatemala will need to increase the rate of decline in childhood deaths from 75.8 to 25.3 deaths per 1000 to achieve the MDG goal by 2015 [4]. To assist member countries with achieving MDG4, the WHO constructed the first Essential Medicines List for Children (EMLc) and launched the “Make Medicines Child Size” effort in December of 2007. This initiative aims to improve the accessibility of safe, effective and quality medicines for children by promoting awareness and action through research, regulatory measures and changes in government policy.

A vital first step to improving medicine access for children is measuring the availability and prices of essential medicines present on pharmacy shelves and in national medicines lists. Data on medicine stock deficits and unaffordable prices creates an evidence base to inform the development of national and regional policies. A recent survey of essential medicines for children in Africa exposed the severe lack of medicines for children in central medicine stores, hospitals, pharmacies and national medicine lists in 14 different countries [5]. The objective of this study is to measure access to children’s medicines in Guatemala using validated methods, similar to those used in Africa [6,7].

### Sources of medicines in Guatemala

The Ministry of Health network is a publically funded group of primary, secondary and tertiary healthcare centers that render services to approximately 70% of the population in Guatemala [8]. Tertiary care hospitals provide a variety of medical specialty and outpatient services and offer medicines on an inpatient basis only, free of charge. The proportion of families who utilize these services or seek treatment elsewhere to care for their sick children is unknown.

The Ministry of Health also operates PROAM, which are pharmacies that offer medicines from *Lista Basica,* a formulary of medicines for common diseases afflicting the population. PROAM was created in 1998 by the Guatemalan Ministry of Health to ensure equal access to quality medications at reasonable prices [9]. The Ministry of Health establishes “*contrato abierto*”*,* a system for setting suppliers and prices in which the government negotiates contracts with pharmaceutical companies to purchase medications [10,11]. The government then distributes medicines from *Lista Basica* needed to stock PROAM pharmacies which are subsequently sold to consumers. PROAM consists of approximately 514 outpatient medicine outlets throughout the country [12]. The law requires that all medications sold must comply with the United States pharmacopeia XXIV quality standards.

The most numerous medicine outlets in Guatemala are privately owned pharmacies, which number close to 4043 establishments throughout the country [12]. This makes the private sector an important source of medicines in Guatemala. There are various laws governing the structure and sanitation of all pharmacies, but no laws regulate the acquisition or prices of medications in the private sector [13-15]. Other sources of medication in Guatemala include Social Security hospitals, private hospitals, dispensing doctors, non-governmental organizations (NGOs) and unregistered dispensaries.

## Methods

We conducted a survey of the availability, prices and affordability of children’s medicines in Guatemala using an adaption of the standardized methodology developed by the World Health Organization (WHO) and Health Action International (HAI) [7]. A validation study of the WHO/HAI survey methodology conducted in Peru in 2005 found that focusing on commonly used medicines yields sufficient and valid results [16]. We collected data on the availability and final patient prices of medicines in a subsector of public and private sector medicine outlets during April and May of 2010.

### Selection of medicine outlets

We selected six regions as survey areas for data collection: Guatemala City, Escuintla, Jalapa, Quetzaltenango, Retalhuleu, and Alta Verapaz. The major urban center of Guatemala City was selected and an additional five “departamentos” or states were chosen at random from those which could be reached within a day’s travel from Guatemala City. These departamentos are all comprised of a collection of urban and rural areas, however, the actual survey areas within each departamento varied demographically.

In each survey area, four to seven private and PROAM sector medicine outlets were randomly selected from a list of pharmacies registered by the Ministry of Health. The actual number surveyed was limited by accuracy of the Ministry of Health registry list and available manpower to carry out the surveys. The private sector sample included 29 outlets and the PROAM sector included 21 outlets. A tertiary public hospital located in each survey area was also included when permission was granted, yielding a sample of four hospitals. Pharmaceutical stores held by private dispensing doctors and non-governmental organizations were not surveyed due to lack of time and resources.

### Selection of medicines

The “Better Medicines for Children Project” specifies a core list of the EMLc to be surveyed, representing medicines commonly used in the treatment of a range of conditions associated with childhood illness prevalent in low income countries [6]. The list specifies the child-specific dosage form and strength for 23 medicines, as well as one device.

We included 22 medicines and one device from the core list in the Guatemala survey. Artemether + lumefantrine was excluded, as it is used for P. falciparum species of malaria, which is not common in the Americas. Primaquine was selected as an alternate malarial medication, as it is effective against P. vivax, the prevalent species of malaria in Guatemala. When alternate formulations were registered in Guatemala, these were added into the list of index medicines and surveyed. In total, 27 medicines and one device were surveyed in Guatemala (Table 1).

**Table 1:** List of medicines surveyed in Guatemala

**No.**	**Disease**	**Name**	**Strength**	**Dosage form**
1	Asthma	Salbutamol*	100 mcg/dose	Inhaler
2	Asthma	Beclomethasone	100 mcg/dose	Inhaler
3	Dehydration	Zinc	20 mg	Dispersible tab
4	Dehydration	Oral Rehydration Solution	500 ml	Solution
5	Dehydration	Oral Rehydration Solution, powder*	To make 500ml	Powder
6	Infectious disease	Amoxicillin	100 mg/ml	Pediatric drops
7	Infectious disease	Amoxicillin	125 mg/5ml	Suspension
8	Infectious disease	Amoxicillin*	250 mg/5ml	Suspension
9	Infectious disease	Amoxicillin + Clavulanic Acid	125 mg + 31.25mg/5ml	Suspension
10	Infectious disease	Amoxicillin + Clavulanic Acid*	250 mg + 62.5mg/5ml	Suspension
11	Infectious disease	Benzathine Penicillin G*	1.2 M units/vial	Injection
12	Infectious disease	Ceftriaxone*	500 mg/vial	Injection
13	Infectious disease	Chloramphenicol	1 g/vial	Injection
14	Infectious disease	Cotrimoxazole (Trimethoprin + Sulfamethoxazole)*	8 + 40 mg/ml	Suspension
15	Infectious disease	Gentamicin*	10mg/ml	Injection
16	Infectious disease	Procaine Penicillin G*	4 M units/vial	Injection
17	Malaria	Primaquine	15mg	Cap/tab
18	Pain/inflammation	Ibuprofen	200 mg	Cap/tab
19	Pain/inflammation	Paracetamol*	25 mg/ml	syrup/susp
20	Pain	Morphine Sulfate	10mg/ml	Injection
21	Xerophthalmia	Vitamin A	50,000 units	Cap/tab
22	Tuberculosis	Isoniazid	100 mg	Cap/tab
23	Anemia	Ferrous Salt*	125 mg/5ml	Suspension
24	Seizure Disorder	Diazepam	5 mg/ml	Injection
25	Seizure Disorder	Carbamazepine	100 mg/5ml	Suspension
26	Seizure Disorder	Phenobarbital	3mg/ml	Oral liquid
27	Seizure Disorder	Phenytoin	25 or 30mg/5ml	Suspension
28	Device – asthma	Spacer	n/a	n/a

* = medicine listed on *Lista Basica.*

For each medicine surveyed, we recorded the highest and lowest price medicine available. In the PROAM sector, manufacturer was also recorded to determine if the product was brand or generic. Medicines in the public hospitals were free of charge to hospitalized patients so only availability was measured.

### Data collection and analysis

Data collection took place during April and May of 2010. Six data collectors were trained according to the WHO/HAI methodology and pilot data collection was conducted. The data collectors visited medicine outlets and collected information on medicine availability and price using a standard data collection form specific to the medicines being surveyed in Guatemala. The data collectors entered survey data into the pre-programmed MS Excel *Workbook* provided as part of the WHO/HAI methodology. Data collection was verified in roughly 10% of pharmacies, data were double-entered and the data checker function on the spreadsheet was used to identify data entry errors.

Three medicines were excluded from the analysis leaving a total of 24 medicines and 1 device for analysis. Phenytoin and phenobarbital suspensions were excluded as the incorrect strengths of the medications were printed on the data collection sheets. Beclomethasone inhaler was omitted from the analysis because although it was included on the core list of children’s medicines for the survey, it was deleted from the EMLc in 2009 [17]. Of note, it was not found in any of the pharmacies surveyed in Guatemala. We calculated the availability of individual medicines as the percentage of sampled medicine outlets where the medicine was found. Data are reported in aggregate and by private vs. public sector pharmacies. We also report mean (average) availability for the list of medicines surveyed.

#### Cross-country comparisons

To facilitate cross-country comparisons, medicine prices obtained during the survey were expressed as ratios relative to a standard set of international reference prices:

$$Medicine\cdot Price\cdot Ratio\cdot (MPR)\cdot =\cdot \frac{median\cdot local\cdot unit\cdot price}{international\cdot reference\cdot unit\cdot price}$$

Medicine price ratios were calculated only for medicines with price data from at least 4 medicine outlets. The exchange rate used to calculate MPRs was 1 US$ = 8.1513 Quetzales; this was the commercial “buy” rate taken from OANDA.com on the first day of data collection [18].

We used the 2009 Management Sciences for Health (MSH) reference prices, taken from the International Drug Price Indicator Guide [19]. These reference prices are the medians of recent procurement prices offered for generic products by for-profit and not-for-profit suppliers to international not-for-profit agencies.

#### Affordability

We assessed the affordability of treating eight of common conditions causing high rates of morbidity and mortality of children in Guatemala by comparing the total cost of medicines prescribed at a standard dose to the daily wage of the lowest paid unskilled government worker at 56 quetzales ($6.87 USD per day) at the time of the survey. For acute conditions, treatment duration was defined as a full course of therapy, while for chronic diseases, the affordability of a 30-days' supply of medicines was determined. Though it is difficult to assess true affordability, treatments costing one days' wage or less were considered affordable [7].

This study was approved by the University of California, San Francisco Committee on Human Research, approval number H2758-35862.

## Results

### Availability of medicines on the day of data collection

As shown in Table 2, the availability of individual medicines varied by type of medicine and sector. Average availability of all surveyed medications was very low. Public tertiary hospitals had the highest availability of medicines found with an average of 46% (range 28-56%) of medicines found in each outlet. Average availability in the private sector was 35% (range 10-52%) for lowest priced medicines. Average availability for a second, higher priced medicine when a lower price medicine was found, was 12% in the private sector.

**Table 2:** Availability of lowest price medications by sector

	**Public hospital**	**Public PROAM**	**Private**
Medicines not found in any	Amoxicillin pediatric drops	Amoxicillin pediatric drops	Chloramphenicol injection
outlets *	Amoxicillin suspension	Amoxicillin suspension	Ferrous Salt suspension
	125mg	125mg	Gentamicin injection
	Amoxicillin-Clav	Amoxicillin-Clav	Isoniazid tablets
	suspension 125mg	suspension 125mg	Morphine Sulfate
	Amoxicillin-Clav	Carbamazepine	injection
	suspension 250mg	suspension	Primaquine tablets
	Carbamazepine	Chloramphenicol	Zinc dispersible tablets
	suspension	Injection	
	Ibuprofen tablets	Diazepam Injection	
	Salbutamol (Albuterol)	Ibuprofen tablets	
	inhaler	Isoniazid tablets	
	Spacer	Morphine Sulfate	
		injection	
		Primaquine tablets	
		Spacer	
		Zinc dispersible tablets	
Medicines found in less than 25% of outlets	Primaquine tablets	Ferrous salt suspension	Amoxicillin pediatric
	Vitamin A tablets	Oral Rehydration	drops
	Zinc dispersible tablets	Solution	Amoxicillin-Clav
		Vitamin A tablets	suspension 250mg
			Carbamazepine
			suspension
			Diazepam injection
			Spacer
			Vitamin A
Medicines found in 25 to 49.9% of outlets		Ceftriaxone Injection	Amoxicillin suspension
		Gentamicin injection	125mg
		Procaine Penicillin G	Amoxicillin suspension
		Salbutamol (Albuterol)	250mg
		Inhaler	Amoxicillin-Clav
			suspension 125mg
			Procaine Penicillin G
Medicines found in 50 to 74.9% of outlets	Amoxicillin suspension	Amoxicillin suspension	Benzathine Penicillin G
	250mg	250mg	injection
	Chloramphenicol	Amoxicillin-Clav	Ibuprofen tablets
	Injection	250mg suspension	Oral Rehydration
	Isoniazid tablets	Cotrimoxazole	Solution, powder
	Oral Rehydration Solution	(Trimethoprim + Sulfamethoxazole	Salbutamol (Albuterol) Inhaler
		Paracetamol (Acetaminophen)	
Medicines found in 75% or more of outlets	Benzathine Penicillin G	Benzathine Penicillin G	Ceftriaxone injection
	injection	injection	Cotrimoxazole
	Ceftriaxone Injection	Oral Rehydration	(Trimethoprim +
	Cotrimoxazole	Solution, powder	Sulfamethoxazole)
	(Trimethoprim +		Oral Rehydration
	Sulfamethoxazole)		Solution
	Diazepam Injection		Paracetamol
	Ferrous salt suspension		(Acetaminophen)
	Gentamicin Injection		
	Morphine Sulfate		
	Injection		
	Oral Rehydration		
	Solution, powder		
	Paracetamol (Acetaminophen)		
	Procaine Penicillin G		

*in the PROAM sector, the Lista Basica determines the medications stocked in the pharmacy. Medications not found in any PROAM outlets were medications not on the Lista Basica.

The PROAM sector had the lowest availability at 25% (range 5-43%). In the PROAM sector, generics were the predominant product type available, with 98% (142/145) of medicines found as generics. Although we expected that only medicines found on the *Lista Basica* would be stocked, some of these medicines had low availability including ferrous salt suspension (24%), gentamicin injection (33%) and procaine penicillin G (29%). When we limited the analysis to the 11 survey medicines found only on the *Lista Basica* (Table 1), availability increased to 57% (range 9-82%) in the PROAM sector. The availabilities of individual medicines in the PROAM, public tertiary hospitals and private sectors are shown in Table 3.

**Table 3:** Availability of individual medicines in the public, PROAM and private sector

	**Percentage of outlets where medicine was found**			
	**Public sector (n = 4 outlets)**	**PROAM sector (n = 21 outlets)**	**Private sector (n = 29 outlets)**	
**Medicine name**	**Lowest price medication**	**Lowest price medication**	**Highest price medication**	**Lowest price medication**
Amoxicillin Pediatric Drops	0.0%	0.0%	6.9%	20.7%
Amoxicillin Suspension 125mg	0.0%	0.0%	6.9%	34.5%
Amoxicillin Suspension 250mg	50%	61.9%	6.9%	48.3%
Amoxicillin-Clav Suspension 125mg	0.0%	0.0%	10.3%	41.4%
Amoxicillin-Clav Suspension 250mg	0.0%	57.1%	3.4%	13.8%
Benzathine Penicillin G Injection	75%	90.5%	20.7%	69.0%
Carbamazepine Suspension	0.0%	0.0%	3.4%	20.7%
Ceftriaxone Injection	100%	47.6%	27.6%	82.8%
Chloramphenicol Injection	50%	0.0%	0.0%	0.0%
Cotrimoxazole (Trimethoprim + Sulfamethoxazole)	100%	57.1%	51.7%	89.7%
Diazepam Injection	75%	0.0%	0.0%	13.8%
Ferrous salt suspension	75%	23.8%	0.0%	0.0%
Gentamicin Injection	100%	33.3%	0.0%	0.0%
Ibuprofen	0.0%	0.0%	13.8%	58.6%
Isoniazid	50%	0.0%	0.0%	0.0%
Morphine Sulfate Injection	75%	0.0%	0.0%	0.0%
Oral Rehydration Solution	50%	9.5%	69.0%	96.6%
Oral Rehydration Solution, powder	100%	81.0%	0.0%	72.4%
Paracetamol (Acetaminophen)	75%	71.4%	41.4%	82.8%
Primaquine	25%	0.0%	0.0%	0.0%
Procaine Penicillin G	100%	28.6%	3.4%	27.6%
Salbutamol (Albuterol) Inhaler	0.0%	42.9%	34.5%	62.1%
Spacer	0.0%	0.0%	3.4%	13.8%
Vitamin A	25%	9.5%	0.0%	13.8%
Zinc	25%	0.0%	0.0%	0.0%

### PROAM and private sector patient prices

As shown in Table 4, lowest price medicines are generally sold at 1.73 times their international reference price in the PROAM sector. Half of the lowest priced generic medicines were priced at 1.24 (25^th^ percentile) to 2.32 (75^th^ percentile) times their international reference price, showing moderate variation in medicine price ratios across individual generic medicines in the PROAM sector.

**Table 4:** Median price ratios of lowest priced medicines, private and PROAM sector patient prices

**Medicine name**	**Lowest price medication MPR(25**^**th**^**-75**^**th**^**percentile)**	
	**Private sector**	**PROAM sector**
Amoxicillin Suspension 125mg	18.86 (14.65-19.54)	n/a
Amoxicillin Suspension 250mg	13.88 (9.60-19.55)	1.85 (1.56-2.04)
Amoxicillin-Clav Suspension 125mg	6.13 (4.50-6.13)	n/a
Amoxicillin-Clav Suspension 250mg	2.53 (2.16-2.76)	0.60 (0.53-0.70)
Benzathine Penicillin G Injection	35.54 (19.33-41.78)	4.51 (3.16-5.86)
Carbamazepine Suspension	3.04 (2.70-3.37)	n/a
Ceftriaxone Injection	13.20 (7.65-18.70)	1.61 (1.10-1.65)
Cotrimoxazole suspension	10.32 (6.42-16.48)	1.37 (1.20-1.82)
Diazepam Injection	19.70 (16.08-26.26)	n/a
Gentamicin Injection	n/a	2.77 (2.08-3.46)
Ibuprofen	31.54 (30.07-36.08)	n/a
Oral Rehydration Solution, powder	5.01 (4.38-7.51)	1.88 (1.25-2.75)
Paracetamol (Acetaminophen)	6.26 (4.72-7.65)	1.05 (0.89-1.70)
Procaine Penicillin G	10.46 (9.15-12.30)	2.46 (2.04-2.65)
Salbutamol (Albuterol) Inhaler	4.94 (3.49-5.56)	1.20 (0.94-1.21)
Vitamin A	15.13 (12.45-15.58)	n/a

* Medicine price ratios (MPRs) were calculated only for medicines with price data from at least 4 medicine outlets.

In contrast, substantial variation exists in median price ratios in the private sector. We compared prices between lowest and highest priced products for six medicines for which more than one price option was found. In the private sector, higher price medications cost twice as much, on average, as their lowest priced equivalents. Higher priced medicines were 22.67 times more expensive than their international reference price. The cost of half of these medicines ranged from 11.21 (25^th^ percentile) to 35.51 (75^th^ percentile) times their international reference price. Lowest price medicines were 10.46 times their international reference price with half of these medicines priced at 5.57 (25^th^ percentile) to 16.99 (75^th^ percentile) times their international reference.

### Comparison of patient prices in the PROAM and private sectors

Median price ratios were substantially higher in the private sector compared to the PROAM sector. Nine medicines that were found in both PROAM and private sectors had data available for price comparisons. As the median MPR was 1.61 and 10.32 for PROAM and private sectors, respectively, final patient prices were 6.41 times higher in the private sector than in the PROAM sector.

### Affordability of standard treatment regimens

As shown in Table 5, lowest price medicines in the private sector were less affordable than in the PROAM sector for most conditions, with standard treatment costing a days' wage or more. Medicines costing over a days’ wage include ceftriaxone injection for susceptible infection costing 15 days wages and carbamazepine suspension for seizure disorders costing 6.6 days wages.

**Table 5:** Affordability: Number of days' wages of worker making lowest paid government wages needed to purchase standard treatments

**Disease condition and ‘standard’ treatment**			**Day’s wages to pay for treatment**	
**Condition**	**Drug name, strength, dosage form**	**Treatment schedule**	**Lowest price medication - Private Sector**	**Lowest price medication - PROAM sector**
Respiratory Tract Infections, UTIs	Amoxicillin Suspension 125 mg/5 mL	Child up to 10 years: 125mg (=5ml) x 3 x 7 days = 105 ml	1.3	n/a
Respiratory Tract Infections, UTIs	Amoxicillin Suspension 250 mg/5 mL	Child over 10 years: 250mg (=5ml) x 3 x 7 days = 105 ml	1.3	0.2
Respiratory Tract Infections, Otitis Media	Amoxicillin-Clavulanic Acid Suspension 125–31.25mg/5 ml	Child 1–6 years: 125mg (=5ml) x 3 x 7 days = 105 ml	2.2	n/a
Respiratory Tract Infections, Otitis Media	Amoxicillin-Clavulanic Acid Suspension 250–62.5mg/5 ml	Child over 10 years: 250mg (=5ml) x 3 x 7 days = 105 ml	2.6	0.6
Seizure Disorder	Carbamazepine Suspension 100mg/5ml	Maintenance treatment: 5mg/kg x 18 kg* x 3 x 30 days = 8100mg or 405 mL for 1 month.	6.6	n/a
Infections due to Susceptible Organisms	Ceftriaxone 500 mg vial	Child under 50 kg: Maximum 1 gram daily x 7 days.	15	1.8
Infections due to Susceptible Organisms	Cotrimoxazole	18 kg x 4mg/kg = 72 mg TMP x 2 x 7 days = 1008 mg. 126 ml total for 7 days.	0.8	0.1
Dehydration	Oral Rehydration Solution, powder to make 500 mL	Moderate Dehydration: 75mL/kg x 18 kg = 1350	0.1	n/a
Pneumonia	Procaine Penicillin G	50mg/kg x 18 kg = 900mgx10 days = 9000mg	1.7	0.4
Pain/inflammation	Paracetamol 24mg/ml suspension	5 year old child: 15mg/kg x 18 kg x 4 x 3 = 3240 mg (=130 mL)	0.5	0.1
Asthma	Salbutamol 100 mcg/dose inhaler	1 inhaler of 200 doses	1.3	0.3
Xerophthalmia	Vitamin A 50,000 units	Child 1–12 years: 200,000 units x 3 doses	0.4	n/a

*Weight of average 5 year old child in Guatemala = 18 kg [31,32].

## Discussion

The availability of essential children’s medicines is low in both the public and private sectors in Guatemala. Availability is lowest in the public sector, but there is inconsistent pricing and poor affordability of medicines in the private sector. Furthermore, formulations of medications that are preferable for use in children were often hard to find. Of the 30 medicine formulations in the survey protocol, seven were not assessed because they are not registered in Guatemala [6]. These consisted of dispersible (3) and chewable (2) tablets, oral liquid (1) and intrarectal solution (1); all formulations preferred for use in children. It is unclear if the lack of registered medicines for children is related to low prescriber demand similar to the case with magnesium sulfate in Zambia, or exclusion from the *contrato abierto*, the central government purchasing mechanism, due to high purchasing prices [20]. Guatemala is subject to rules and regulations under Trade Related Aspects of Intellectual Property Rights (TRIPS). Essential medicines that have been reformulated to be better suited for children may be under patent and therefore more expensive in Guatemala for up to 5 to 15 years after becoming generic in the United States [11]. Another factor that may be responsible for a dearth of registered child friendly formulations may be difficultly obtaining the product if it is not manufactured in the country or nearby. As suggested in previous literature, studies of local prescribing practices and government purchasing are required to fully answer these questions [5]. Development of a national essential medicines list that includes children’s medicines could increase demand from providers and guide purchasing decisions [21].

Among the surveyed medicines, originator brands were almost never available in the PROAM sector as the government contracts with generic pharmaceutical companies to procure less expensive medications. In addition, the availability of generic medicines in the PROAM sector was exceptionally low with only about one quarter of index medicines stocked in each outlet. These findings are similar to a recent study on the accessibility of children’s medicines in 14 African countries, where essential children’s medicines were available at central medicine stores 15 to 75% of time with only 3 medicine outlets containing over 50% of medicines [5]. Our findings are also consistent with a number of pricing and availability surveys that have been conducted for adult medicines for both acute and chronic diseases [22-25]. These studies show that availability of essential medicines is lower in the public than the private sector, varies by country, and that medicines for chronic conditions are less available than those for acute conditions.

Seven medications were not found at all in the private sector. One medication, chloramphenicol injection, was not stocked due to concerns about its toxicity. One would expect the remainder of these medicines to be found at the hospital level and therefore, not stocked in outpatient outlets. However, as the availability in hospitals is low, friends or family of hospitalized patients often go to alternate locations such as private retail pharmacies to purchase medications. Even the poorest patients in Guatemala seek medicines from the private sector [26].

Diarrheal disease is one of the major causes of mortality in children under five in Guatemala [8]. Although oral rehydration powders for reconstitution and premixed solutions for treatment of diarrhea were available in the PROAM and private sectors, oral zinc was unavailable in these sectors and the mean availability was only 25% in the hospital setting. Zinc is included on the EMLc due to its benefit in treating children with diarrhea [27].

Antibiotics for treating pneumonia and other respiratory infections in children should be available for use as empiric treatment, to decrease a major cause of morbidity in children [8]. While ceftriaxone was found quite frequently in the private and public sectors, it was only available in about half of the PROAM outlets. Amoxicillin and amoxicillin-clavulanic acid were available most frequently in 250mg/5ml concentration. When child appropriate concentrations are not available, pharmacy employees must calculate the dose. This could lead to adverse drug events as pharmacy employees in low income countries may need additional training [28].

In the PROAM sector, affordability of lowest price medicines was reasonable compared to the private sector where many of lowest priced treatments cost more than the daily wage of a lowest paid government employee. In the private sector, the most unaffordable treatments were for seizure disorder with carbamazepine (6.6 days wages) and infections with ceftriaxone (15 days wages). Carbamazepine is an ongoing treatment for a chronic condition, making it even less affordable. Given that 24% of the population in Guatemala is living below the international poverty line of less than $2/day, treatments which appear affordable may still be too costly [29]. In addition, treatment costs refer to medicines only and do not include the additional costs of consultation and diagnostic tests. Finally, families who need medications for more than one child may be confronted with insurmountable drug costs. These findings are consistent with other studies of affordability of adult medicines showing that chronic medicines, in particular, are unaffordable for many populations [22,23,25,30].

A major strength of this study is the use of a previously validated methodology which allows for the measurement of medicine prices and availability in a reliable and standardized way [16]. Additional strengths include training and utilization of multiple check points to ensure quality data collection, data entry and interpretation.

Limitations of the WHO/HAI methodology are consistent with past surveys. Therapeutic alternatives or alternate dosage forms were not assessed. The availability data refers to the day of data collection at each particular facility in subsectors of the country and may not reflect average monthly or yearly availability of medicines at individual facilities or throughout the entire country. Also, the standard for determining the median price ratio is the median of supplier prices. However, if supplier prices are not available, buyer prices are substituted to determine the median international reference price for the different medications. This can lead to artificially high or low reference price that isn’t necessarily indicative of true median price paid internationally [25]. NGOs, private dispensing doctors and unregistered dispensaries were not surveyed and their importance as a source of medicines for those with low socioeconomic status is unknown. Further investigation is warranted into the amount that these sources contribute to accessibility to medications in Guatemala.

## Conclusion

The findings of this study can be used to structure policies to improve the accessibility of children’s medicines in Guatemala and help the country meet MDG4 in 2015. Monitoring the availability and pricing of children’s essential medicines in Guatemala and other countries is crucial for improving transparency about global prices. Updated lists of all registered pharmacies are needed to facilitate these analyses. Inclusion of the WHO’s Essential Medicines List for children into national formularies and purchasing lists such as the PROAM and *contrato abierto* could improve the availability of essential children’s medicines. Data should be collected on prescribing practices of local pediatricians to detect deficiencies, if any, in rational prescribing and develop educational programs to improve prescribing of medicines from the EMLc [5]. The coordination of the WHO EMLc with national clinical practice guidelines could promote physician use of essential medicines. Further analysis of the procurement, supply and distribution of specific medicines is needed to determine reasons for lack of availability [20].

## Competing interest

The authors declare that they have no competing interests.

## Author’s contributions

AA, BR, ARE and LB conceived of the study, and participated in its design and coordination and helped to draft the manuscript. All authors read and approved the final manuscript.

## Author’s information

AA is a PharmD and a Pharmacy Practice Resident at University of San Francisco, California Medical Center. BR is a PharmD and a Health Sciences Assistant Clinical Professor at University of San Francisco, California and a Professor in the Departamento de Química-Farmacéutica at the Universidad del Valle de Guatemala. ARE is an MPH and a consultant at Cedar Associates. LB is a PhD and Professor of Clinical Pharmacy and Health Policy.
